# Using the Change Manager Model for the Hippocampal System to Predict Connectivity and Neurophysiological Parameters in the Perirhinal Cortex

**DOI:** 10.1155/2016/8625875

**Published:** 2015-12-27

**Authors:** L. Andrew Coward, Tamas D. Gedeon

**Affiliations:** Australian National University, Canberra, ACT 0200, Australia

## Abstract

Theoretical arguments demonstrate that practical considerations, including the needs to limit physiological resources and to learn without interference with prior learning, severely constrain the anatomical architecture of the brain. These arguments identify the hippocampal system as the change manager for the cortex, with the role of selecting the most appropriate locations for cortical receptive field changes at each point in time and driving those changes. This role results in the hippocampal system recording the identities of groups of cortical receptive fields that changed at the same time. These types of records can also be used to reactivate the receptive fields active during individual unique past events, providing mechanisms for episodic memory retrieval. Our theoretical arguments identify the perirhinal cortex as one important focal point both for driving changes and for recording and retrieving episodic memories. The retrieval of episodic memories must not drive unnecessary receptive field changes, and this consideration places strong constraints on neuron properties and connectivity within and between the perirhinal cortex and regular cortex. Hence the model predicts a number of such properties and connectivity. Experimental test of these falsifiable predictions would clarify how change is managed in the cortex and how episodic memories are retrieved.

## 1. Introduction

In order to perform behaviours, a system requires information processing resources. The way in which these resources are organized is called the system architecture. An overall system architecture for the brain which could be mapped into major anatomical structures was originally proposed in Coward [1]. This architecture, called the recommendation architecture, can provide an account for a wide range of cognitive phenomena in terms of anatomically plausible processes.

**Figure 1:**
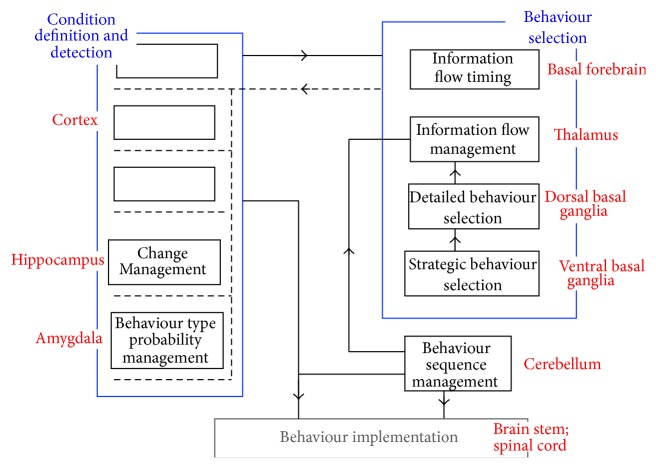
Brain system architecture that results from the natural selection pressures exerted by a range of practical considerations. Considerations like the need to limit physical resources and the need to learn without interference with prior learning tend to constrain the physical architecture into the illustrated form, with different subsystems performing different information processing functions. The correspondence between the predicted subsystems and the different major anatomical structures of the mammal brain is shown.

If a system needs to perform a complex combination of behaviours, practical considerations place severe constraints on the system architecture. The considerations include the need to limit the total information processing resources required, the need to modify some behaviours without undesirable side effects on other behaviours, and the need to build systems with the minimum of construction errors. In the case of electronic systems designed under external intellectual control, it can be demonstrated that these considerations lead to the presence of data read/write and instruction information processes, a separation between memory and processing subsystems that specialize in the two types of process, and the sequential execution of instructions [2]. In the case of a system which must learn a complex combination of behaviours, it can be demonstrated that these considerations lead to the recommendation architecture. The major features of this recommendation architecture are presence of condition definition/detection and behavioural recommendation processes, a separation between two major subsystems performing condition definition/detection and behaviour selection processes, and the layering of condition definition/detection resources [2]. More specifically, it can be demonstrated that as illustrated in Figure 1 the two major subsystems tend to be subdivided in specific ways, and there is an additional major subsystem that records sequences of behaviours that are frequently used and have already been learned [3].

The recommendation architecture illustrated in Figure 1 is not an absolute requirement for a complex learning system. Rather, it is a limit towards which the architecture of a complex learning system will tend, as the practical considerations become more strict. When the ratio of the number of behaviours to be learned to the available information processing resources increases, the system architecture will be constrained more and more into this architectural form. Because natural selection will favour brains that can learn a given set of behaviours with fewer resources, it can be predicted that brains required to learn a high proportion of their behaviours will be strongly constrained into Figure 1 architecture. Comparisons between this recommendation architecture and a wide range of other cognitive models demonstrate that it is better able to support higher cognitive processes in a plausible fashion [2, 4]. There is extensive psychological, anatomical, and physiological evidence that the mammal brain has been constrained into this architecture [3, 5] and that the different predicted architectural subsystems correspond with the major anatomical structures of the mammal brain, as illustrated in Figure 1.

Cortical pyramidal neurons heuristically define receptive fields, where the term receptive field can be generalized from its traditional sensory definition to mean the information circumstances in which a pyramidal neuron produces an output. A pyramidal neuron gets inputs from a large group of other pyramidal neurons, these inputs establishing synapses on its dendritic tree. Pyramidal neurons have complex dendritic trees, typically with of the order of 100 terminal branches [6, 7] which determine the electrical responses of the dendrites [8]. Each terminal branch typically has of the order of 100 synapses from other pyramidal neurons. Passage of action potentials over the surface of the dendrite communicates the effects of different terminal branches [9] resulting in integration of their effects [10]. In information terms, a branch can therefore be regarded as a set of conditions defined by its synapses. If enough synapses on one branch are active, a condition is detected. If enough conditions are detected across different branches, the receptive field of the neuron is detected. This integration arrangement is illustrated in Figure 8.

In the recommendation architecture model, one receptive field can acquire recommendation strengths in favour of a wide range of different behaviours. In other words, the detection of a receptive field indicates the presence of some circumstance, and the recommendation weight in favour of one behaviour indicates the probability that the behaviour is appropriate when that circumstance is present. The basal ganglia determine which behaviour has the highest recommendation weight across all currently detected receptive fields and implement that behaviour. The striatum in the basal ganglia is where most cortical inputs arrive, and neurons in the striatum correspond with very specific behaviours [11]. A recommendation weight is therefore instantiated by the synaptic strength of a cortical neuron on to the striatal projection neuron that corresponds with the behaviour.

The complexity of a receptive field can be roughly defined as the number and type of raw sensory inputs that contribute to its definition, either directly or via intermediate receptive fields. Receptive fields on different levels of complexity will be more effective for recommending different types of behaviours. As a result, the cortex is organized into different areas, each area receiving inputs from a small group of other areas and therefore detecting receptive fields in a different range of complexity [12].

Receptive field changes implemented by synaptic weight changes are the physical substrates for learning [13, 14]. Receptive fields must therefore change over time. However, receptive field changes also present a major problem. Any receptive field possesses existing behavioural meanings, so any changes to the field jeopardize the integrity of these meanings. This is essentially the catastrophic interference problem observed in artificial neural networks [15]. As a result, receptive field changes must be very tightly managed. In particular, changes must only be made when necessary and must have as little effect on existing recommendation weights as possible, generally meaning that they must be as small as possible.

These constraints on change have a number of implications [16].* Firstly*, since receptive field detections recommend behaviours, if a cortical area is receiving inputs and is only detecting a small number of receptive fields, the number of recommendations will be too small for a high integrity selection. The criterion for changes being necessary is therefore that the number of detections in an area is below some minimum limit.* Secondly*, qualitative changes to receptive fields will mean that the existing recommendation strengths will be inappropriate. Hence the only types of change can be small expansions to the range of circumstances in which the field is detected or a general increase in the detection threshold if the field is being detected too often. It could also be possible to eliminate synaptic components to a receptive field if those components never contribute to receptive field detections.* Thirdly*, since receptive field changes must be as small as possible, some way must exist to determine how close undetected fields are to detection. If additional detections are required, the fields closest to detection can then be expanded. These three implications specify the form that change management takes in the brain.

## 2. The Hippocampal System

There is a very structured pattern of connectivity between the hippocampus, a small group of cortical areas, and parts of a number of subcortical structures. In this paper this group of structures and the connectivity between them will be called the hippocampal system. It corresponds to a degree with what in older terminology was called the limbic system.

In more detail, this hippocampal system is defined as the hippocampus proper (dentate gyrus, CA fields, and subicular complex) and three cortical areas closely associated with hippocampus proper (the entorhinal, perirhinal, and parahippocampal cortices) plus parts of a range of subcortical structures. All the rest of the cortex, except for the primary sensory areas, is reciprocally connected with the three cortical areas. The full system is illustrated in Figure 4.

Coward [1] originally proposed that, within the framework of the recommendation architecture, the hippocampus managed changes to cortical receptive fields, determining when and where such changes occur. A more detailed model for how the full structure illustrated in Figure 4 performs cortical change management has been extensively developed since then [16, 17] and compared with the neuroscience and cognitive predictions it makes and with a range of alternative models [16].

## 3. Change Management in the Cortex

In this recommendation architecture based model for managing changes to receptive fields across the cortex, a primary role of cortical columns is to make it possible to identify which currently undetected cortical receptive fields are good candidates for expansion when such expansions are needed. This is achieved because,* firstly*, all the neurons in a column detect relatively similar receptive fields [18].* Secondly*, there is a pattern in the flow of connectivity through a column. Inputs arrive in layer IV, and neurons in layer IV project to neurons in layers II/III. Neurons in layers II/III project to layers V/VI which are the outputs from the column. Hence the layer V/VI neurons can be regarded as detecting the receptive field of the column. The layering and connectivity mean that neuron receptive fields in layers II/III are slightly more complex than in layer IV and are therefore detected slightly less often. Receptive fields in layers V/VI are even more complex and are detected even less often.

As a result, if there is no activity by layer V/VI neurons in a column, the column is not detecting its receptive field. If in this situation there is strong activity in layers II/III, the implication is that the column is fairly close to detecting its receptive field and would be a good candidate for receptive field expansion if an increase in the number of detections is required.

The three problems to be solved are then,* firstly*, how to determine if receptive field expansions are needed,* secondly*, how to identify the columns with the largest internal activity, and,* thirdly*, how to drive expansions in those identified columns. As proposed in Coward [1, 16], the solution is provided by the hippocampal system.

The hippocampal system identifies the most appropriate columns on the basis of two criteria: firstly, the degree of internal column activity; secondly, if a column has often expanded its receptive field in the past at the same time as a number of columns that are already producing outputs. To implement this second criterion, the hippocampal system records groups of cortical columns that have expanded at the same time in the past and constantly updates these receptive fields with each expansion episode.

Neurons in layers II/III all across the cortex except the primary sensory cortices project to the perirhinal and parahippocampal cortices [19]. Layers II/III in these cortices project to the entorhinal cortex [20], and layers II/III in the entorhinal cortex project to the hippocampus proper [21]. Hence the hippocampal system gets information about the internal activity of columns all across the cortex. Connectivity in the opposite direction most heavily targets the deeper cortical layers.

When a mammal is at a specific physical location, it can be expected that many receptive fields in different parts of the cortex will expand at the same time. Some neurons in the hippocampal system detect their receptive fields when the brain is at specific locations that are different for each such neuron [22, 23]. The existence of such neurons is evidence supporting the general model that hippocampal system receptive fields correspond with groups of cortical columns that expanded their receptive fields at the same time in the past.

The primary role of these hippocampal system receptive fields is to drive current receptive field expansions. However, some of the fields may also be used for other purposes, such as navigation [24].

The receptive fields in the hippocampus proper and the associated cortical areas are thus defined by groups of cortical columns that expanded their receptive fields at similar times in the past. Going from the perirhinal and parahippocampal cortices to the entorhinal cortex and then to the hippocampus proper, the groups of columns included in a receptive field definition become larger, and the columns come from more cortical areas.

As described in more detail below, within CA3 and the dentate gyrus in the hippocampus proper, several feedback loops identify the degree of novelty in current sensory inputs and therefore the degree of receptive field expansion required. This circuitry also identifies the large groups of cortical columns where receptive field expansions are most appropriate. Once this identification process is complete, the selections are released to CA1 by the action of the septal nuclei. From CA1 they are decoded through the subicular complex, the entorhinal cortex, and the perirhinal and parahippocampal cortices. This decoding results in the targeting of neurons in the most appropriate columns to drive receptive field expansions.

## 4. Accessing the Recommendation Strengths of Cortical Receptive Fields

Cognitive tasks are accomplished by accessing the recommendation weights associated with cortical receptive fields. This access requires activation of the neurons and columns that record these receptive fields, and these activations are communicated to the basal ganglia where the recommendation weights are stored. Activation of receptive fields occurs as a result of their presence in current sensory inputs, but complex cognitive tasks require access to a much wider range of information than is available in these directly detected receptive fields. Accessing this wider range of information requires indirect activation of receptive fields that are not being detected within current sensory inputs. Because of constraints on resources, any one receptive field is associated with multiple cognitive concepts like semantic object categories or episodic events. For example, any one receptive field is detected within the presence of instances of multiple different types of visual object [18]. Hence cognitive information cannot be used to guide such indirect activations. The only type of information the cortex can use to carry out the indirect activations needed to support cognitive tasks is past temporal correlations in the activity of anatomical structures like neurons or columns.

Five types of memory were identified by Schacter and Tulving [25] on the basis of a range of criteria. These types, of memory systems, are working, priming, semantic, episodic, and procedural memory. There are four types of temporal correlation that are possible, and each type results in one of the different types of memory [3, 17]. The first is prolongation of the activity of receptive fields that are already being detected. Indirect activation on this basis is the primary information mechanism supporting working memory. The second type is indirect activation of inactive receptive fields on the basis of recent past activity at the same time as some currently active receptive fields. Indirect activation on this basis is the primary information mechanism supporting priming memory. The third type is indirect activation of inactive receptive fields on the basis of frequent past activity at the same time as some currently active receptive fields. Indirect activation on this basis is the primary information mechanism supporting semantic memory. The fourth type is indirect activation of inactive receptive fields on the basis of activity during a past receptive field expansion episode at the same time as some currently active receptive fields. Indirect activation on this basis is the primary information mechanism supporting episodic memory. In the recommendation architecture model, procedural memory, the fifth type of memory identified by Schacter and Tulving [25], depends upon changes to the weights of synapses made by cortical neurons on to striatal neurons. In information terms these change the recommendation weights associated with cortical receptive field detections.

It is important to note that if an episodic memory is frequently recalled, the information mechanism could shift to indirect activation on the basis of frequent past simultaneous activity. In other words, frequent recall can change a memory from episodic to semantic, resulting in different cortical structures supporting the recall. For example, when a word is first learned there can be recall of the circumstances in which it was learned, but later, only the word meaning can be recalled.

Uncontrolled indirect activations could lead to a chaotic pattern of activity. Indirect activations must therefore be managed as behaviours. These indirect activation behaviours must be recommended by cortical receptive field detections and accepted or rejected by the basal ganglia just as for any other behaviour type.

One neuron could be directly activated by sensory inputs or indirectly activated on the basis of past temporally correlated activity with other neurons. To avoid inappropriate activations, a neuron must therefore have at least two separate receptive fields, one defined by a combination of sensory circumstances and the other defined by combinations of activities by a range of other cortical columns indicating that indirect activation is appropriate. In general, the direct receptive field is defined on the basal dendrites and the indirect receptive field on the apical dendrite [26] (see Figure 8).

## 5. The Hippocampal System and Episodic Memory Retrieval

An episodic memory is a partial reexperience of a unique earlier event. Such a reexperience requires activation of a receptive field population that is similar to the population active during the event. Almost all events have a degree of novelty that will result in receptive field expansions. As proposed in Coward [1], indirect activation on the basis of past simultaneous expansion means that a seed population of columns containing some that were active during such an event could drive activation of a more complete subset of the event population.

Hippocampal receptive fields record the kind of information that would be needed to drive indirect activation of inactive receptive fields on the basis of simultaneous past receptive field expansion. The hippocampal system is known to play a role both in the creation of new declarative memories and in the retrieval of episodic memories. Damage to the hippocampal system results in loss of the ability to create new semantic and episodic memories (e.g., [27]), as would be expected to result when the ability to expand cortical receptive fields is lost. In addition, the hippocampal system also plays a key role in episodic memory retrieval [28] and damage can also result in the loss of ability to retrieve some episodic memories, suggesting that it also has a role in the required indirect activations [27]. For some patients there is a loss of episodic memory for events that occurred during a period of number of years prior to hippocampal damage, but some earlier events can be recalled [29]. However, in the same patient, semantic memories created in the same period can be recalled [30]. This is consistent with a shift from recall on the basis of past simultaneous receptive field expansion to recall based on frequent past simultaneous activity for episodic memories that are often recalled.

However, there are two problems with using the primary information recorded in the hippocampal system for episodic memory retrieval. Firstly, if the same fields used to drive receptive field expansions are also used to drive indirect activations, such indirect activations will tend to result in unnecessary expansions. Secondly, an episodic memory needs to activate a substantial proportion of the receptive fields active during the earlier experience, not just the fields that expanded. Solving these two problems requires hippocampal receptive fields that are separate from those driving receptive field expansions and correspond with all the cortical receptive fields that were active during past periods of receptive field expansions, not just the fields that expanded.

This implies a need for at least two types of neuron level receptive fields in the hippocampal system. One type will correspond with groups of cortical columns that expanded at similar times in the past, and the outputs from these neurons will drive receptive field expansions. The second type will correspond with groups of cortical columns that were active during past periods of receptive field expansion, and the outputs from these neurons will drive indirect activations.

The interface between most of the cortex as a whole and the hippocampal system is the perirhinal and parahippocampal cortices [31]. Hence receptive fields corresponding with groups of columns with the two types of temporal correlations in past activity can be expected to be present in those cortical areas.

## 6. Detailed Model for Hippocampal System Management of Receptive Field Expansion and Episodic Memory Retrieval

**Figure 2:**
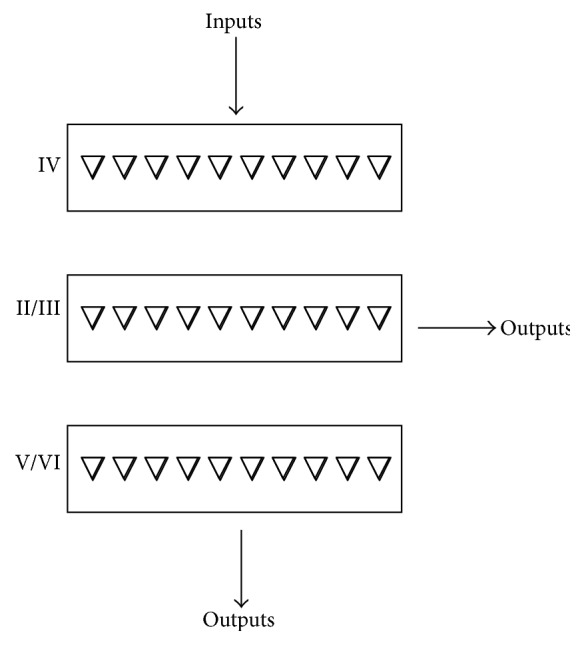
Cortical column structure drawn by predominant connectivity flow rather than physical arrangement. Inputs to a column arrive in layer IV, outputs from IV go to layers II/III, and outputs from layers II/III go to layers V/VI. Outputs from layers V/VI are the outputs from the column. Because receptive fields in one layer are combinations of receptive fields in the preceding layer, they are more complex and specific and will therefore be detected somewhat less often on average than receptive fields in the preceding layer. If there is activity in layers V/VI, the column is already producing an output and no receptive field expansion is appropriate. However, if there is strong activity in layers II/III but no output, this indicates that the column is fairly close to detecting its receptive field. Because only a slight expansion would be needed to get detection, such expansion would be relatively low risk.

The anatomical model based on these considerations is summarized in Figures 2 through 9.  Figure 2 is a simplified model for a cortical column, with the five usual layers of pyramidal neurons simplified to three. The primary connectivity route is that inputs from outside the column arrive in layer IV, projections from layer IV go to layers II/III, projections from layers II/III go to layers V/VI, and layers V/VI are the primary outputs from the column. Neuron receptive fields in layers V/VI are therefore slightly more complex than those in II/III and those in layer IV the least complex. Hence as described earlier, activity in layers II/III is the internal activity of the column that can be used to recommend receptive field expansions.

**Figure 3:**
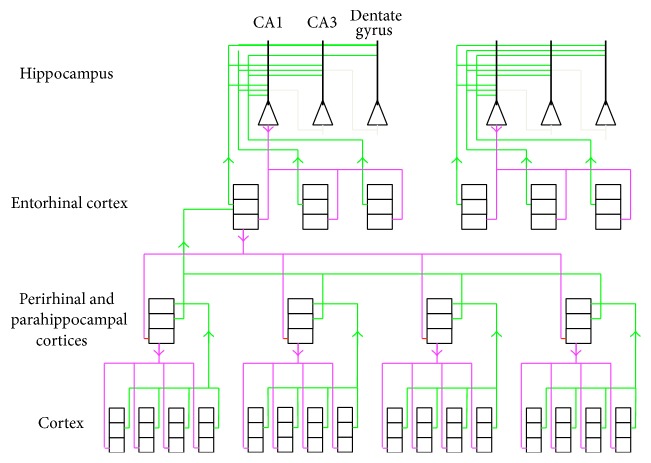
Internal layer II/III activity of columns across the cortex is transmitted through the perirhinal, parahippocampal, and entorhinal cortices to the hippocampus proper. Columns in these hippocampal system cortical areas develop receptive fields corresponding with groups of cortical columns that tended to expand at similar times in the past. A competition in the hippocampus proper identifies the most appropriate groups of columns for current receptive field expansion. Signals then go back through layers V/VI of the columns in the selected groups, driving column receptive field expansions.

**Figure 4:**
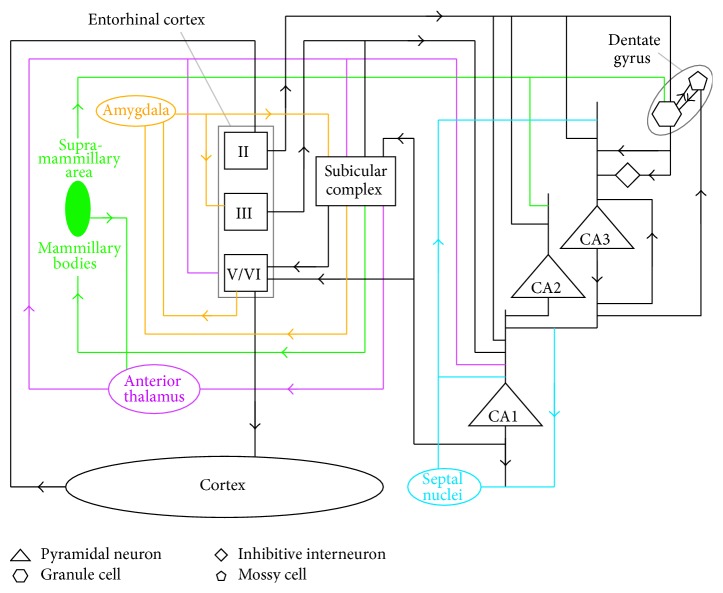
The full hippocampal system model. In addition to the flow of connectivity from across the cortex to the hippocampus proper and back to the cortex, there are a number of subcortical structures that play functional roles. The thalamus has the role of releasing cortical activity between areas by placing gamma band frequency modulations on neuron spike outputs. CA1 drives receptive field expansions on the basis of inputs from CA3. While the competition to select cortical column groups for expansion is under way, the inputs from CA3 do not reflect final selections. CA3 outputs are therefore only released to CA1 when the septal nuclei detect that the competition has concluded. The release is then carried out by imposition of an additional theta band modulation. The mammillary bodies act on the competition process to bias the degree of receptive field expansion in favour of cortical regions that recommend behaviours of the general type that is currently the priority. The amygdala acts on the results of the competition process, to increase the degree of receptive field expansions in more complex cortical areas that record information about the gist of the situation. See Coward [3, 16] for more details.

As illustrated in Figure 3, receptive fields throughout the hippocampal system correspond with groups of cortical columns that expanded their receptive fields at the same time. A number of very large groups of columns from all across the cortex are selected as expansion targets by the hippocampus proper, and as a result of the decoding process through the associated cortices, columns that appear in a number of these very large groups will have a high chance of receiving signals that will drive their expansion. Subcortical structures also play roles in the hippocampal system. These roles are summarized in Figure 4 [3].

**Figure 5:**
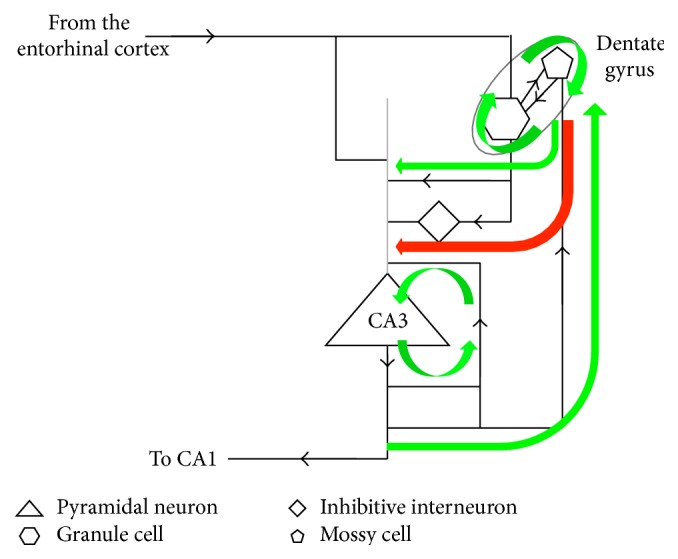
Competition circuitry within the hippocampus proper to select cortical columns for receptive field expansions. Mossy cells in the dentate gyrus target CA3 pyramidal neurons with similar receptive fields, directly which is excitatory and in an inhibitive manner via interneurons. As the activity of the mossy cell increases, inhibition becomes predominant. If the situation being processed by the cortex is familiar, there will be strong input from the entorhinal cortex corresponding with all relevant cortical areas. This will mean strong activity of all relevant granule cells, and the predominant effect of these cells on all the CA3 pyramidal neurons will be inhibitive. If there is novelty in some aspects of the current situation, some granule cells will be less active, allowing CA3 activity to develop. Activity of CA3 neurons corresponding with the cortical areas needing receptive field expansions will develop, and as it develops it will be self-limited because it drives increased activity in the dentate gyrus. So the CA3 activity will be proportional to the degree of novelty in the current experience and will be targeted on the cortical columns most appropriate for the necessary expansions to respond to the novelty. Once the competition process is complete, CA3 drives CA1 activity, which sends signals back to the cortex to drive expansions.

Figure 5 shows the connectivity within the hippocampus proper. There are two positive feedback loops: excitatory connectivity from CA3 pyramidal neurons to CA3 pyramidal neurons [32] and reciprocal excitatory connectivity between granule cells and mossy cells in the dentate gyrus [33]. There is a positive feedback loop between these two feedback loops: excitatory connectivity from CA3 pyramidal neurons to dentate gyrus mossy cells [34–36] and from dentate gyrus granule cells to CA3 pyramidal neurons [37]. Finally, there is an inhibitory path from granule cells via interneurons to CA3 pyramidal neurons. At relatively low levels of granule cell activity, its excitatory outputs to CA3 predominate over the inhibitory path via interneurons. As granule cell activity increases, the inhibitory path begins to predominate [38].

The functional role of this connectivity proposed in Coward [16] is as follows. If there is little novelty in the current cortical inputs, there will be high levels of receptive field detections in all cortical areas. The entorhinal cortex inputs to all dentate gyrus granule cells will therefore be high, and the inhibitory path from granule cells to CA3 will predominate, eliminating CA3 activity. Absence of CA3 activity will mean no CA1 outputs and therefore no cortical receptive field expansions. If there is some novelty in current cortical inputs, some cortical areas will detect a relatively low level of receptive fields. The entorhinal cortex inputs derived from those areas will therefore be lower, and the activity of dentate gyrus granule cells with receptive fields including those areas will be lower. This will allow buildup of CA3 pyramidal neurons with receptive fields including those areas. The positive feedback loop within CA3 will focus CA3 activity on receptive fields with the best match to the cortical areas with lower activity. As CA3 activity builds, the feedback to the granule cells via the mossy cells will increase, increasing the inhibition of CA3. The rise in CA3 activity is therefore self-limiting and will be proportional to the degree of novelty in current cortical inputs [39]. Once the activity has reached an equilibrium, the CA3 activity will correspond with the most appropriate large groups of cortical columns for expansion, and this activity is released to CA1 to initiate decoding through the subicular complex and the three cortical areas associated with the hippocampus. This decoding results in activity targeting the most appropriate columns for current expansion.

**Figure 6:**
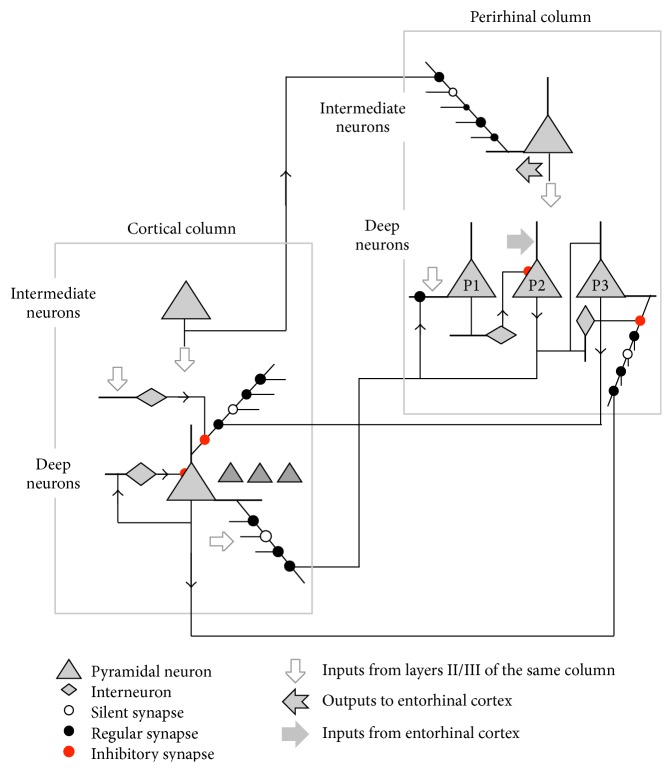
Neuron level connectivity between perirhinal cortex and sensory cortex area. This connectivity supports change management and episodic retrieval. See text for explanation.

The perirhinal and parahippocampal cortices are the main interface between the hippocampal system and much of the rest of the cortex. Hence a key aspect of the model is to define what is happening at this interface. The model for this interface at the level of neuron connectivity between the perirhinal cortex and regular cortex is shown in Figure 6. In the figure, two columns are illustrated. In each column just a few deep neurons (layers V/VI) and one intermediate neuron (layers II/III) are shown. The connectivity is illustrated for just a few example neurons.

The cortical column in Figure 6 could be located anywhere in the cortical areas that interface with the perirhinal cortex. The direct receptive field of this column is some combination of sensory inputs, possibly multimodal. This column receptive field is defined on the basal dendrites of the deep neurons in the column. These basal dendrites receive most of their input synapses from intermediate layer II/III neurons in the same column, which in turn get inputs from layer IV. The column can also be indirectly activated. In this case, the deep neurons detect an indirect receptive field defined on their apical dendrites. It is important to note that the same cortical deep neurons are activated directly as a result of a sensory experience or indirectly on the basis of past temporally correlated activity during a period of receptive field expansion. There will therefore be similarities in the subjective experiences.

The receptive field of the perirhinal column in Figure 6 includes the illustrated cortical column. However, the inputs defining the perirhinal receptive field come from the intermediate layers of the cortical column and of course many other cortical columns. These inputs arrive on the basal dendrites of the intermediate neurons of the perirhinal column. The illustrated cortical column will in general form part of the receptive field of many other perirhinal columns. In the perirhinal column there is a requirement for three populations of deep neurons, the P1, P2, and P3 populations illustrated. The role of the P1 population is to detect the regular receptive field of the column, defined by strong activity in the intermediate neurons of the column. The role of the P2 neurons is to drive receptive field expansions in appropriate circumstances. These circumstances are indicated by strong inputs from the entorhinal cortex. Such inputs mean that the group of regular cortical columns defining the receptive field of the perirhinal column have been selected by the hippocampal system for current receptive field expansion. The role of the P3 neurons is to record the group of deep neurons in the cortical columns that are active during a period of receptive field expansion. P3 neurons can then drive indirect activations in appropriate circumstances.

If there is input to the cortical column derived from sensory inputs, there could be activity in the intermediate neurons which could drive receptive field detections by the deep column neurons through their basal dendrites. The intermediate activity is also communicated to the intermediate neurons in the perirhinal column. These intermediate perirhinal neurons target the entorhinal cortex and through the entorhinal cortex the hippocampus proper. The intermediate neurons in the perirhinal column also target the P1 population of deep neurons. If these P1 neurons detect their receptive fields, this indicates that the degree of activity across the cortical columns making up the receptive field of the perirhinal column is large enough that receptive field expansions are probably not appropriate. The P1 neurons therefore inhibit the P2 neurons via interneurons.

If the hippocampus proper selects the group of cortical columns defined by this perirhinal column as a good target for receptive field expansion, signals from the entorhinal cortex arrive on the P2 population. If not inhibited by P1 activity, firing of the P2 population will encourage receptive field expansions of the basal receptive field of the deep cortical neurons and also of the P1 population. These P2 outputs drive activation of the P3 population on their apical dendrites and may also drive receptive field expansions in the basal dendrites of the P3 population and the apical dendrites of the cortical deep neurons (connectivity not illustrated in Figure 6). Receptive field expansions may perhaps also be driven by P2 outputs to the intermediate neurons.

The deep neurons in the cortical column target the basal dendrites of the P3 population, and the P3 neurons target the apical dendrites of the deep neurons in the cortical column. This is the connectivity that supports indirect activations. In a period of receptive field expansion, some P3 neurons will be active, driven by P2 outputs. Some cortical deep neurons will also be active, with or without receptive field expansions. The P3 neurons will record on their basal dendrites the groups of deep cortical neurons active during the period, and the deep cortical neurons will record on their apical dendrites the groups of P3 neurons active during the period. Note that the P3 neurons will record groups of deep neurons from multiple cortical columns, and the deep neurons in a cortical column could record a group of P3 neurons in multiple perirhinal columns.

This connectivity established during a period of receptive field expansion means that if a small subset of the set of deep cortical neurons active during the period of expansion were activated later and their activity released to the P3 population, the returning P3 outputs would tend to activate a larger proportion of the set. For example, if the subset was activated as a result of hearing some trigger words, this mechanism could lead to activation of a high proportion of the neurons active during the original experience.

There are a couple of other connectivity paths needed to maintain the integrity of these mechanisms. During a period of receptive field expansion in response to sensory inputs, the deep cortical neurons need to detect their receptive fields only within their inputs from intermediate cortical neurons. The P3 population needs to be active and will be providing inputs to the apical dendrites. The effect of these inputs on deep cortical neuron firing must be blocked. In the figure, this blocking is achieved by interneurons that target the point where apical inputs enter the soma. These interneurons are activated by the intermediate neurons in the column, which will be active when inputs derived from sensory sources are present, but not when indirect activations are under way. In the perirhinal column during a receptive field expansion period, the contribution of the indirect activation basal dendrite receptive field is blocked by interneurons that are targeted by the P2 neurons.

**Figure 7:**
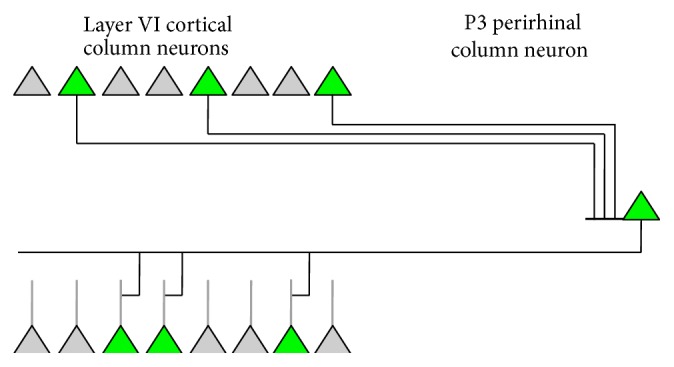
Delayed firing neurons and sequential access of sequential episodes of an episodic memory. This connectivity creates the ability to move through the memory of an event in temporal sequence. The recall of one moment drives recall of the next moment.

Hence this circuitry supports both receptive field expansions and indirect activations for episodic retrieval, each in the appropriate circumstances. Three populations of deep neurons are indeed observed in the perirhinal cortex: regular spiking; burst spiking; and late spiking [40]. This fits well with the proposed model. The P1 population is detecting a normal column receptive field and would correspond with the regular firing neurons. The P2 population is driving receptive field expansions, and the burst firing would be effective for this purpose (see discussion of neuron mechanisms below). The P3 population is late firing, with delays up to more than 10 seconds. This could support retrieval of a sequence of times in an episodic memory. For example, a late firing P3 neuron is illustrated in Figure 7. This neuron has provisional, perhaps zero weight, synaptic inputs from and outputs to cortical deep column neurons. If the upper group of deep cortical neurons were active during a period of receptive field expansion at some point in time and the lower group at a slightly later point in time, then the upper group could establish strong connectivity on to the P3 neuron when they were active, and when the P3 neuron fired a little later, it could establish strong connectivity on to the lower group. The effect would be that if the P3 neuron was later activated in response to the upper group, it could drive activation of the lower group. In other words, the recall of a situation at one point in time could drive recall of a situation at a slightly later point in time.

**Figure 8:**
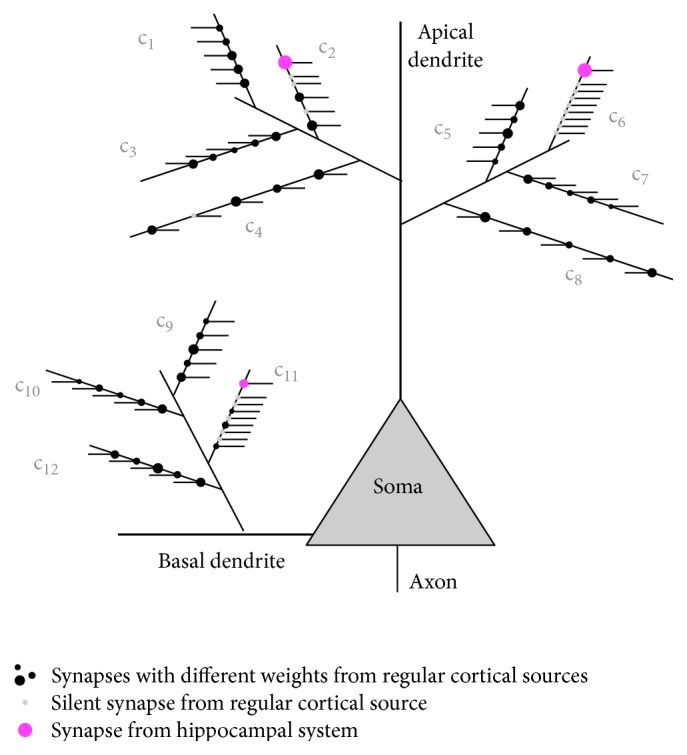
The receptive fields of a neuron. The basal dendrites define a receptive field within sensory input states. The apical dendrite defines a receptive field indicating when indirect activation of the neuron is appropriate. Conditions are defined by synapses on dendritic terminal branches, and a receptive field is detected if a significant proportion of the branches detect their conditions. In general, only one receptive field can contribute to neuron firing at one point in time.

The model for a receptive field at the neuron level is illustrated in Figure 8. The basal and apical dendrites record different receptive fields. Generally the basal dendrites detect a receptive field within the current sensory input state, and the apical dendrite detects a receptive field that defines the circumstances in which the neuron should be indirectly activated. Individual conditions are defined by groups of synapses from other neurons on to one dendritic terminal branch. If a branch receives sufficient inputs within a fairly short period of time, it injects potential deeper into the dendrite. This injection may be initiation of a dendritic calcium action potential and indicates a condition detection by the branch. If sufficient branches detect their conditions, the receptive field is detected. Integration by basal and apical fields is separate, and in general only one will determine neuron firing at any point in time.

**Figure 9:**
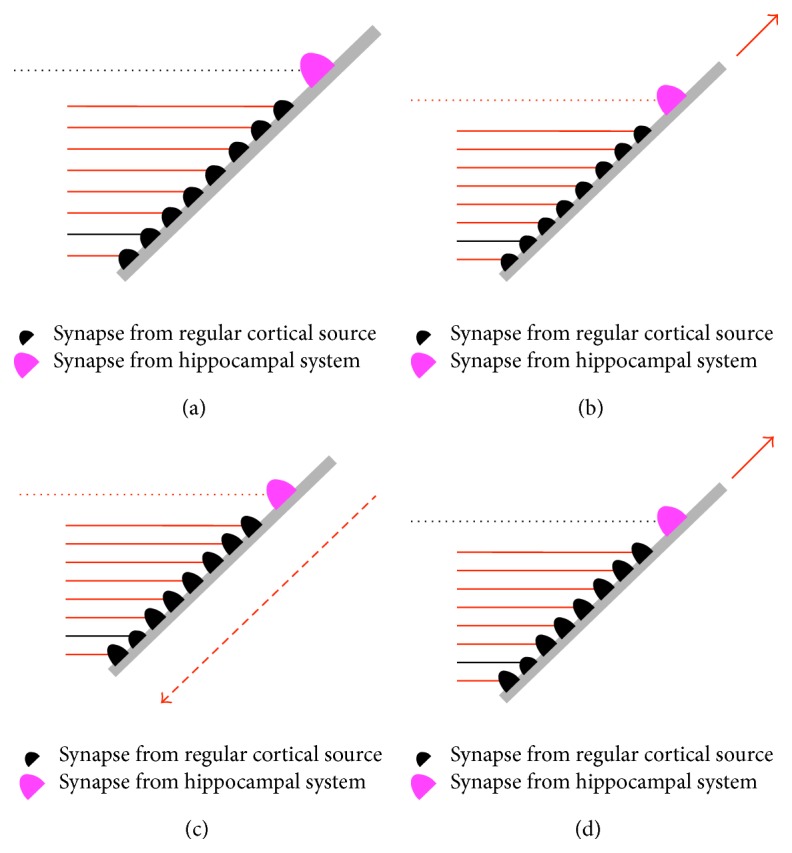
Process for hippocampal system managed receptive field expansion at dendritic terminal branch level. (a) Regular cortical synapses on the branch have insufficient total weight to cause the branch to inject potential deeper into the dendrite; (b) activity of the hippocampal system input causes injection of dendritic potential; (c) if the neuron fires, a backpropagating action potential increases the weights of recently active cortical synapses; (d) the weight increases mean that the branch in the future can inject potential into the dendrite, independent of the hippocampal system input.

Receptive field expansions increase the range of circumstances in which the receptive field is detected. Such expansions are implemented by changes to conditions, and these changes are influenced by the hippocampal system. The process for heuristic definition of conditions under hippocampal system control is conceptually illustrated in Figure 9. The branch illustrated in Figure 9(a) has a set of relatively weak, perhaps even silent synapses from regular cortical sources. Even if all of the synapses are receiving inputs, there is insufficient injected postsynaptic potential to trigger injection of potential deeper into the dendrite. Hence this branch does not contribute to receptive field detection in any circumstances. However, there is also a strong input from the perirhinal cortex P2 population to the branch. If the hippocampal system is selecting neurons in this column for receptive field expansion, this input is active as illustrated in Figure 9(b). In this situation there is sufficient synaptic input to generate a branch output. If shortly afterwards the neuron fires, the backpropagating action potential increases the weights of all the recently active regular cortical inputs (Figure 9(c)). As a result, as shown in Figure 9(d), these regular cortical inputs now have enough total weight that their future activity could result in injection of potential deeper into the dendrite, independent of hippocampal system inputs. Effectively, a new condition has been recorded on the neuron.

Figure 9 illustrates a relatively extreme case, recording of a completely new condition. In some cases a hippocampal input to a branch with some already strong synapses could significantly increase the weights of weaker synapses and even slightly increase the weights of stronger synapses. In some neurons, such as the P3 population discussed earlier, P2 could act by inputs to the apical dendrite causing the neuron as a whole to fire, increasing the weight of recently active synapses on branches of the basal dendrite.

## 7. Relationship of Model with Cognition, Anatomy, and Physiology

As reviewed in Coward [16], there is extensive cognitive, anatomical, and physiological evidence for the proposed model. However, at the more detailed level developed in this paper, the model makes a number of more specific predictions which could be tested by experiment. These predictions fall into a number of categories including physical connectivity, neuron dynamics, and correlations of neuron activity with behavioural circumstances.

### 7.1. Physical Connectivity

*Prediction 1.* In the model, the P2 population of burst spiking neurons drives receptive field expansions on the basis of inputs from higher in the hippocampal system. The prediction is therefore that most inputs from the entorhinal cortex to the perirhinal cortex will terminate on the burst spiking neurons. It has already been observed that the burst firing neurons have much more pronounced apical dendrites than the two other types [40], which is consistent with more inputs from the higher entorhinal area.

*Prediction 2.* In the model, the receptive field of a column is defined by the P1 population of regular spiking neurons. The prediction is therefore that most of the targets of layer II/III neurons within a perirhinal cortex column target will be the regular spiking neurons.

*Prediction 3.* The model predicts that projections from layer II/III neurons to deep neurons within a regular cortical column should predominantly synapse on the basal dendrites of the deep neurons.

*Prediction 4.* The model predicts reciprocal connectivity between deep neurons in regular cortical columns and the P3 population of delayed spiking neurons in the perirhinal cortex. The P3 population is predicted to synapse mainly on the apical dendrites of the deep cortical neurons in regular cortical neurons, while the reciprocal projection is predicted to synapse mainly on the basal dendrites of the P3 delayed spiking perirhinal neurons.

*Prediction 5.* The model predicts that the population P1 regular spiking deep neurons in the perirhinal cortex will inhibit the P2 burst spiking neurons via interneurons.

*Prediction 6.* The model predicts that the population P2 burst spiking deep neurons in the perirhinal cortex will directly excite the P3 population of delayed spiking neurons by targeting their apical dendrites but will inhibit the basal dendrites of the P3 neurons via interneurons.

*Prediction 7.* The model predicts that the layer II/III neurons in a regular column will directly excite the deep neurons by targeting their basal dendrites but will inhibit the apical dendrites of the deep neurons via interneurons.

### 7.2. Neuron Dynamics

*Prediction 8.* The model predicts that synapses made by P2 population burst firing perirhinal neurons will have high initial weights or be multiple, but the weights will decline over time.

*Prediction 9.* The model predicts that backpropagating action potentials in deep cortical neurons will increase the weights of recently active synapses on to branches in the apical dendrite that have recently initiated a dendritic action potential towards the soma, even if the contribution of the apical dendrite to neuron firing was blocked from actually reaching the soma by inhibitory synapses.

### 7.3. Activity Correlations with Behaviours

*Prediction 10.* The model predicts that interneuron activity will ensure that, for deep neurons in live behaviour, either basal or apical inputs can contribute to neuron firing, but not both.

*Prediction 11.* The model predicts that there will be more activity of burst firing deep perirhinal neurons during novel experiences.

## 8. Conclusions

The understanding of the hippocampal system as the change manager for the cortex is supported by a wide range of cognitive, anatomical, and physiological evidence. The model proposed earlier [16] can be extended to a more detailed level. This extension leads to a number of predictions of the most probable connectivity and neuron algorithms within and between the perirhinal cortex and regular cortical areas. Experimental investigation of these falsifiable predictions would be very valuable for clarifying understanding of the operation of the hippocampal system.
